# Edaravone reduces depression severity in patients with symptomatic intracranial stenosis and is associated with the serum expression of sex hormones

**DOI:** 10.1097/MD.0000000000019316

**Published:** 2020-02-21

**Authors:** Zhaohong Kong, Jian Jiang, Ming Deng, Zhaohui Zhang, Gaohua Wang

**Affiliations:** a Institute of Neuropsychiatry and Mental Health Center; b Department of Neurology, Renmin Hospital of Wuhan University, Wuhan, China.

**Keywords:** depression, edaravone, sex hormone, stenting, symptomatic intracranial arterial stenosis

## Abstract

**Objective:**

To investigate the effect of edaravone on depression relief in symptomatic patients with intracranial stenosis and its relationship with the expression of sex hormones.

**Methods:**

We recruited 112 patients with symptomatic intracranial arterial stenosis from Renmin Hospital, Wuhan University, between October 2014 and October 2017. All patients were divided into the traditional or experimental (traditional treatment + intravenous infusion of edaravone 30 mg twice a day for 14 days) treatment groups. The general clinical data were collected, and neurological functional recovery using the Modified Rankin Scale (mRS) and National Institute of Health stroke scale (NIHSS) scores were recorded. Symptom Checklist 90 (SCL-90) was used to assess the general psychological changes of the patient, followed by the 24 Hamilton Depression Scale (HAMD) to examine the incidence of post-stroke depression (PSD). This divided the patients into the mild, moderate, and severe depression groups. Next, we measured the serum protein expression of the sex hormones estradiol (E2), testosterone (T), follicle stimulating hormone (FSH), prolactin (PRL), and luteinizing hormone (LH).

**Results:**

The mRS and NIHSS scores were significantly lower in the experimental group than in the control group (*P* < .05). There was no significant difference in SCL90 score before intervention (*P* > .05); the scores were significantly lower in the experimental group after intervention (*P* < .05). There was a significant difference in SCL-90 and HAMD scores between groups before treatment (*P* < .05), with significantly lower scores in the experimental group post-treatment (*P* < .05). The incidence of depression was significantly reduced in the experimental group post-treatment. Furthermore, the expression of E2 and FSH was significantly higher (*P* < .01) and lower (*P* < .001), respectively, in women than in men in the experimental group post-treatment. Interestingly, the expression of T was significantly lower in men in the experimental group post-treatment (*P* < .001).

**Conclusion:**

Edaravone significantly improved the clinical efficacy of stent implantation in intracranial artery stenosis treatment by alleviating depression and reducing the incidence of PSD.

## 1 Introduction

Intracranial artery stenosis or occlusion is a significant risk factor for cerebral ischemic disease in Asian and African Americans.^[1–3]^ Recent studies have suggested that stroke caused by middle cerebral artery (MCA) stenosis accounts for 4% to 15% of all patients per year.^[4–7]^ Endovascular stent angioplasty is a novel treatment for intracranial artery stenosis when compared with drug therapy.^[8,9]^ Some studies have suggested that patients who receive the cerebral artery angioplasty show improved functional outcomes and reduced mortality.^[9]^ However, complications, such as post-stroke depression (PSD), can seriously affect the clinical prognosis, and increase morbidity and mortality.^[10]^ Furthermore, the relationship between depressive status and the expression of sex hormones in patients with intracranial artery stenosis remains unknown.

Edaravone (3-methyl-1-phenyl-2-pyrazole Lin-5-ketone) is a nerve protectant in ischemic stroke, and is domestically recognized in China. Edaravone contains clear hydroxyl free radicals that inhibit lipid peroxides and restrain the oxygen free radicals, thereby attenuating inflammation in the body.^[11]^ Previous studies have assessed the neuroprotective effect of edaravone on acute ischemia reperfusion injury; however, few clinical studies have reported its protective effect following stent implantation. This study sought to explore the effect of edaravone administration on patients with depression following symptomatic intracranial stenosis stent implantation.

## 2 Methods

### 2.1 Participants

G Power was used to perform the sample size calculation for this study. According to a previous study,^[12]^ the effect size for a primary outcome was the occurrence of depression among patients with stroke. A sample size of 56 in each group was required to achieve a type I error of 0.05 with a power of 0.80 in two group analyses of covariance (ANCOVA). Therefore, we recruited 112 patients who underwent intracranial artery stenosis and intracranial stenting in the Department of Neurology between October 2014 and October 2017. Treatment was based on the guidelines of the diagnosis and treatment of acute ischemic stroke, as previously reported.^[13]^

Patients’ clinical data, including age, gender, cardiovascular, and cerebrovascular risk factors, body mass index (BMI), clinical manifestations, revascularization, and adjuvant drug treatment were collected. **Inclusion criteria were**:

1. Symptomatic intracranial artery stenosis, aged 18 to 85 years old, and intracranial angiography showing arterial stenosis ≥70%;
2. stenotic vascular normal diameter ≥2 mm, normal distal vascular diameter, posterior circulation vascular lesion length <20 mm, and length of the anterior circulation vascular lesion <15 mm;
3. no serious neurological dysfunction after stroke;
4. ≥1 risk factors for atherosclerosis, such as hypertension, diabetes, abnormal lipid metabolism, smoking history, obesity, and history of coronary heart disease;
5. no history of recent hemorrhagic disease or disease requiring surgery in the near future;
6. clinical symptoms treated by anti-platelet aggregation for >3 months without positive effect;
7. within 14 days of acute stroke, or ≥3 months after stroke/transient ischemic attack (TIA), there is a stent placement pointer, and the patients provide consent to receive stent placement.

**Exclusion criteria were**:

1. Other intracranial lesions (such as intracranial hemorrhage, aneurysm, and arteriovenous malformation);
2. other causes of arterial stenosis (such as vascular dissection and muscle fiber dysplasia);
3. extreme stenosis or stenosis with difficulty in surgery;
4. severe liver and kidney dysfunction or other serious systemic diseases;
5. age, <18 or >85 years old;
6. previous mental disorders (such as history of anxiety and depression or cognitive dysfunction) and unable to provide consent.

This study was approved by the ethics committee of Renmin Hospital, Wuhan University. We obtained written informed consent from each patient or substitute decision maker.

### 2.2 Treatment

Patients were treated according to standard interventional therapy.^[13]^ Briefly, oral aspirin (300 mg/day) and clopidogrel (75 mg/day) were administered 5 days before surgery. The location, extent, and collateral circulation of the arteries were confirmed based on the angiographic results. The diameter of the stent was selected according to the diameter of the proximal and distal normal vessels. All patients had been administered a routine treatment of aspirin (100 mg/day) and clopidogrel (75 mg/day), and Lipitor (20 mg/day) for 3 months before surgery. Blood pressure was controlled within 130/80 mm Hg and blood glucose control was monitored every 2 weeks. The edaravone group (experimental group) was administered an intravenous infusion of edaravone (30 mg), twice for 3 days after continuous conventional therapy for 14 days.

### 2.3 Neurological function tests

A battery of neuropsychological tests was administered within 7 days before and 4 weeks after the surgery using the modified Rankin Scale (mRS) and National Institutes of Health Stroke Scale (NIHSS). Cognitive function evaluation was performed by two independent clinical psychologists who were blinded to the outcome of the intervention.

### 2.4 Depressed state evaluation

Symptom Checklist 9 (SCL90) was used to evaluate the general psychological stress of patients. This is a common neuropsychology screening scale divided into 5 grades. The composition of the scale includes measurement factor scores for nine symptoms, including somatization, depression, anxiety, interpersonal relationship, obsessive-compulsive symptoms, hostility, terror, and psychotic symptoms. A total score ≥160, ≥43 positive items, or any factor that scores >3 points is considered positive. The scale is suitable for domestic adults in China and has good reliability and validity. A standardized instruction was used by two trained professionals to conduct the self-assessment questionnaires for participants independently. Following this, blinded methods were used to ensure that the quality of each response was valid.

The Hamilton Depression Rating Scale (HAMD)^[15]^ was used to evaluate patients’ depressive state before admission for treatment and at 4 weeks after admission. The two neurological factors were independently assessed by trained psychological clinicians. In addition, the results were checked for consistency. First, the patients were grouped according to education level, including illiterate (uneducated), primary school (≤6 years of education), or middle school and above (>6 years of education). Patients that did not fully complete data collection or did not show reliability were excluded. For patients who were included, depression was scored as follows: mild, 8 to 20; moderate, 21 to 35; and severe, ≥36.

### 2.5 Assessing serum sex hormone expression

We collected 3 mL of blood from the elbow vein with patients who had fasted for. The blood samples were centrifuged immediately. The serum was collected and stored in the refrigerator at −20°C for further testing. Estradiol (E2), testosterone (T), follicle stimulating hormone (FSH), prolactin (PRL), and luteinizing hormone (LH) were measured using an Access LH assay kit (chemiluminescence method), according to the manufacturer's instructions (Beckman Coulter Co., Ltd., USA), using an Access immunoassay system manufactured by Beckman Coulter Co., Ltd., USA. The recovery rate of this method is 99% to 102.2%; the intra-assay coefficient of variation is 3.8% to 5.4%, and the coefficient of variation between batches is 4.3% to 6.4%.

### 2.6 Statistical analysis

Data were collected using Excel software and the statistical analysis was performed using SPSS 19.0 (IBM, New York). Categorical variables were summarized by frequency and percentage. Continuous variables were summarized by mean, median, range, and standard deviation. The sampled population was divided into diagnostic groups of individuals with and without depression. The chi-square and Mann–Whitney *U* tests were used analyze the categorical and continuous variables (that were not normally distributed), respectively, between groups. The paired *t* test was used to compare two groups, and the one-way ANOVA and Kruskal–Wallis test, with post hoc tests for two-group comparisons, was used to compare ≥3 groups. Differences were considered statistically significant when *P* < .05.

## 3 Results

### 3.1 Patients

The patients’ characteristics, including age, gender, cardiovascular, and cerebrovascular risk factors, clinical manifestations, revascularization, and adjuvant drug therapy are presenting in Table 1. There were no significant differences between groups (*P* > .05). Hypertension refers to systolic blood pressure ≥140 mm Hg or diastolic blood pressure ≥90 mm Hg. Hyperglycemia refers to fasting blood glucose ≥6.9 mmol/L after meals and/or blood glucose ≥11.1 mmol/1 h. Dyslipidemia refers to total cholesterol (TC) >5.2 mmol/L, triglyceride (TG) >1.72 mmol/L lipoprotein a (LP a) >300 mg/L, low-density lipoprotein (LDL) >3.1 Methyl/L, an increase in apolipoprotein B (Apo-B) >1.1 g/L, and a meaningful drop in high-density lipoprotein (HDL) <0.9 mmol/L and apolipoprotein A1 (Apo-Al) <1.0 g/L.

**Table 1:**
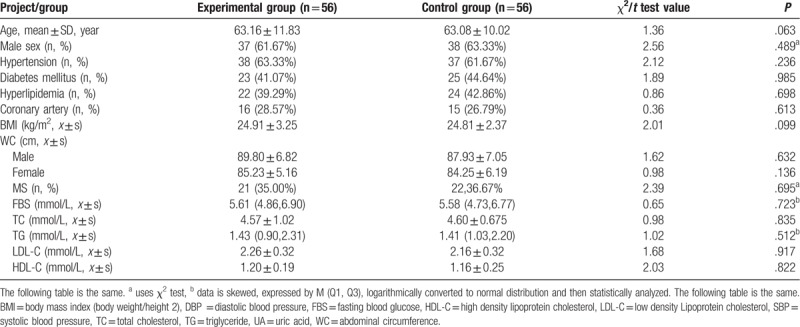
Baseline characteristics of the two groups.

### 3.2 Recovery of neurological function

**Figure 1:**
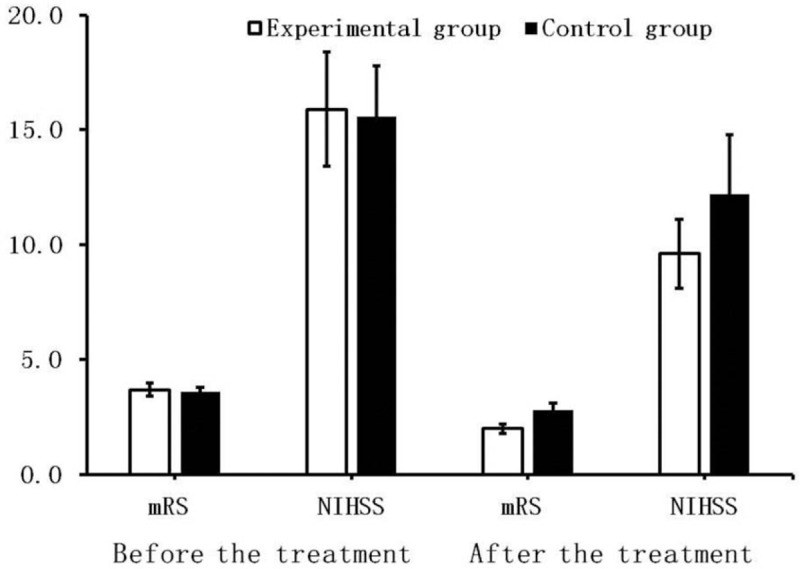
Results of mRS and NIHSS scores in both groups. There are no significant differences in neurological function scores between groups before the treatment. At 4 weeks, there are significant reductions in neurological function scores in the experimental group when compared with the control group (*P* = .036 and .027, respectively).

There was no significant difference between groups in the mRS and NIHSS scores before admission (*P* = .067 and .089, respectively), indicating no clinical difference between the groups of patients before treatment (Table 2, Fig. 1).

**Table 2:**
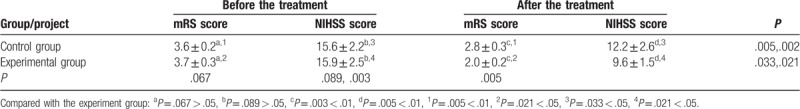
Results of mRS and NIHSS scores in both groups ( ± s, n = 60).

After treatment, there was significant recovery of neurological function in both groups; however, neurological function scores in the experimental group were significantly lower when compared with the control group (*P* = .003 and .005, respectively; Table 2, Fig. 1).

### 3.3 Evaluation of depression between groups

We collected 112 SCL-90 self-assessment questionnaires from participants. This included 9 invalidated questionnaires (experimental group, n = 2; control group, n = 7). There was no significant difference in SCL-90 scores between groups before treatment and (*P* > .05; Table 3).

**Table 3:**
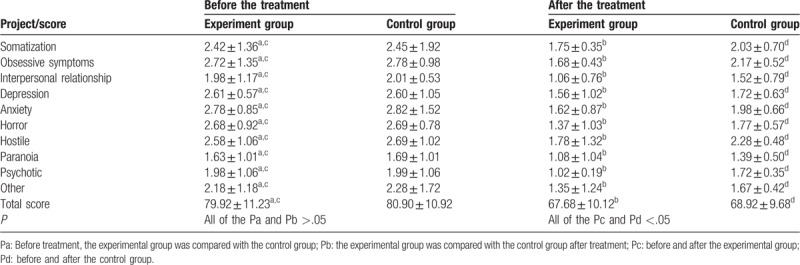
Scl-90 scores of the two groups before and after treatment (*x* ± SD).

At 4 weeks post-treatment, there was a significant reduction in factor scores in both groups (*P* < .05), indicating that the intervention measures after admission were beneficial at alleviating the psychological distress in patients. Further statistical analysis showed that there was a significantly greater reduction in scores in the experimental group when compared with the control group (*P* < .05; Table 3).

To further study the effects of the two treatment methods on the psychological changes in the patients, we evaluated 24 candidates using the HAMD Depression Scale and measured the incidence of mild, moderate, and major depression in both groups (Table 4). Interestingly, there were differences in the incidence of mild-to-moderate depression between groups before treatment; however, this did not reach significance. After treatment, there were significant differences in the incidence of depression between groups (*P* < .05), with a reduction in the severity of depression in the experimental group when compared with the control group (Tables 4 and 5).

**Table 4:**
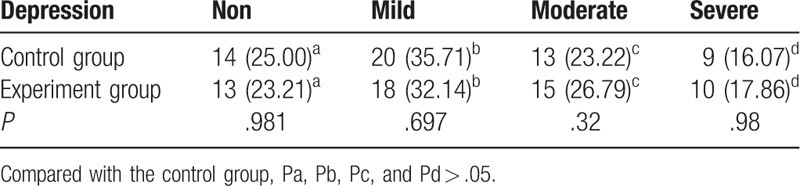
Comparison of depression status before treatment (n [%]).

**Table 5:**
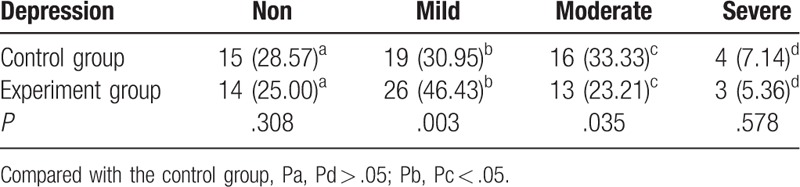
Comparison of the depression status after treatment (n [%]).

### 3.4 Serum expression of sex hormones

Our results revealed significantly higher expression of E2 in females in the experimental group at 4 weeks post-treatment when compared with females in the control group (*P* < .01). Furthermore, the expression of FSH was significantly lower in females in the experimental group at 4 weeks post-treatment when compared with the control group (*P* < .01). There were no significant differences between the expression of T, PRL, and LH in females between groups (*P* > .05; Tables 6 and 7).

**Table 6:**
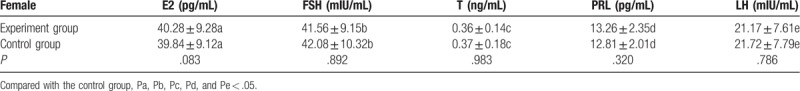
The serum levels of sex hormone in female before the treatment (means ± SD).

**Table 7:**
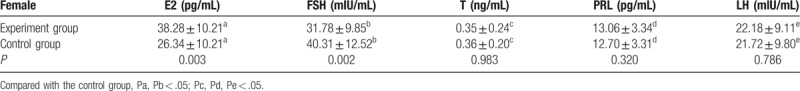
The serum levels of sex hormone in female in 4 weeks (means ± SD).

In males, the serum expression of T was lower in the experimental group when compared with the control group (*P* < .001). There were no significant differences in E2, FSH, PRL, and LH in males between groups (*P* > .05; Tables 8 and 9).

**Table 8:**
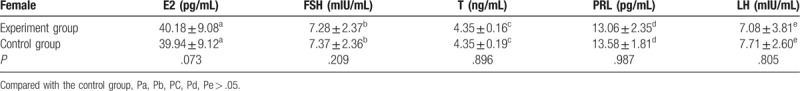
The serum levels of sex hormone in male before the treatment (means ± SD).

**Table 9:**
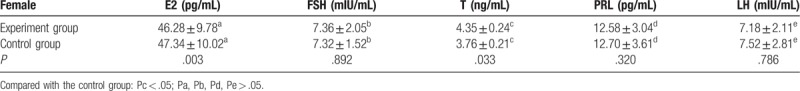
The serum levels of sex hormone in male in 4 weeks (means ± SD).

### 3.5 Safety and adverse reaction assessment

We enrolled 56 patients who were administered edaravone sodium chloride in this study. The patient side effects at 4 weeks post-treatment were reported as follows. One out of 56 patients experienced limb soreness. This appeared at 3 days after treatment, disappeared after 1 day of application observation. No others side effects were reported; therefore, the safety of edaravone was relatively high.

## 4 Discussion

As we all know, there is a strong psychological stress response when individuals suffer from acute trauma. The greater the unpredictability of this event, the stronger the psychological stress response it can cause. This can lead to serious physiological dysfunction affects the prognosis of the patient's physiological function. Reducing this stress can help with disease prognosis and improve the quality of life for patients. In this study, we found no differences in the general clinical data between patients, indicating that the groups of patients are comparable.

We explored the changes in psychological stress in patients with symptomatic intracranial stenosis and the effect of edaravone on their psychological function. First, we used the SCL90 to assess changes in psychological stress. These results showed that there was an increase in psychological assessment scores in all patients, which indicated that stroke had a significant psychological impact on the patients. There was no significant difference between groups before treatment. Following a 4-week period of recovery and treatment, the patient's psychological assessment scores were significantly reduced in both groups, indicating that psychological stress can be improved in patients after hospitalization. There are some possible reasons for this. First, with the patients begin to slowly accept changes in their physiological functions over time and adapt psychologically. Second, recovery of function occurs with disease treatment, which supports the recovery of the patients’ psychological function.

A further depression scale test showed that mild and moderate depression was more common in the two groups before treatment, with no significant difference between groups. However, following a period of hospitalization, the incidence of depression in both groups was significantly reduced, with significantly lower incidence in the group treated with edaravone when compared with the control group, which is consistent with previous results. Psychological changes after trauma may directly or indirectly affect patient prognosis. Therefore, early detection or effective psychological intervention can improve the clinical prognosis of the patient.

PSD is common in patients who experience complications after stroke and affects their clinical prognosis.^[11]^ In this study, we found that administration of edaravone can reduce the severity of depression in patients. There are a number of possible causes underlying this effect. First, edaravone can effectively cross the blood–brain barrier and effectively inhibit the vascular endothelium and cell damage. Edaravone is lipophilic and has a binding rate of 90% to human serum protein and albumin,^[16,17]^ which can reach the brain faster and inhibit lipid peroxidation, thereby inhibiting the delayed onset neuronal death.^[10]^ Second, edaravone scavenges oxygen free radicals, which cause the oxidation of unsaturated fatty acids in brain cell membranes, lysosomal damage, and the aggravation of cognitive and psychological functions.^[18]^ Third, edaravone reduces the high and low shear viscosity, and plasma specific viscosity of patients with cerebrovascular disease, and improve intracranial blood supply status.^[19]^ Fourth, edaravone inhibits the expression of the proapoptotic gene, caspase-3, reduces the degree of apoptosis, and accelerates the recovery of neurological deficits.^[20]^ Fifth, PSD may be influenced by age, gender, stroke type, stroke site, history of depression, and cognitive impairment.^[21,22]^

In addition, other unknown factors may reduce the severity of depression in post-stroke patients. For example, previous studies have shown that edaravone reduces inflammation in patients with cerebral infarction *via* the Akt/mTOR signaling pathway, by reducing the expression of IL-1β and TNF-α, and by reducing damage to the blood–brain barrier.^[23,24]^ Furthermore, chronic low-grade inflammation contributes to the pathophysiological changes associated with major depressive disorder (MDD). Recently, studies have shown that psychological stress can induce an inflammatory response; therefore, inflammation is linked to physical and mental ill health.^[25,26]^ Most of these studies focus on individuals with depression people; therefore, there is little relevant data for patients with ischemic brain damage.^[25]^ The importance of the inflammatory response in stroke patients requires further study.

We analyzed the serum expression of sex hormones to explore the underlying possible mechanism of edaravone. Previous studies have demonstrated that the incidence of PSD in women is higher than that of men.^[27]^ In this study, the expression of E2 and FSH were significantly higher and lower, respectively, in female patients the experimental group when compared with the control group. Conversely, the expression of T in the experimental group was significantly higher than in the control group in male patients. These results indicated that edaravone can improve the severity of depression in patients with symptomatic intracranial stenosis, which may be related to sex hormone expression. Previous studies have reported associations between the expression of men and T, and associations in women with E2 and FSH, which are in line with our results.^[28,29]^ Furthermore, sex hormones have been linked to the development of depression.^[30,31]^ Studies have shown that depression can be improved in women *via* the administration of exogenous sex hormones. In addition, the occurrence of depression in females is associated with a dynamic decline in sex hormone levels.^[32]^ Estrogen exerts its antidepressant effect *via* the 5-hydroxytryptamine (5-HT) system. Briefly, estrogen causes post-synaptic desensitization of the 5-HT1A receptor by upregulating the G protein signaling Z1 (RGSZl).^[33,34]^ Therefore, the age-related decline in E2 in women leads reduces the antidepressant effect. Furthermore, a reduction in E2 leads to an increase in the secretion of FSH, *via* the endocrine feedback mechanism. The expression of T in the male patients with PSD decreased in both groups in our study, in line with a previous by Molly et al.^[35]^ This may be linked to a reduction in T altering the function of 5-HT in the central nervous system and reducing 5-HT2A. The density of the receptor makes the elderly more susceptible to depression.

There are some limitations in this study, such as the lack of long-term continuity, further clinical follow-up, and prospective clinical data. In this study, we collected clinical data from patients at 7 days and 4 weeks post-treatment. Further prospective studies will be carried out to collect clinical data at baseline, and 3, 6, 12, and 24 months after intervention to further evaluate the clinical efficacy of edaravone and its relationship with the sex hormones. In addition, the study lacked further clinical follow-up data, such as sub-group analyses of the specific infarct location, degree of arterial stenosis, and location of the symptomatic intracranial arterial stenosis. Finally, there was a lack of prospective clinical data. However, despite these limitations, edaravone is a commonly used neuroprotective agent in Chinese clinical practice and has important application prospects in patients following arterial stent implantation.

## 5 Conclusion

Edaravone can significantly improve the clinical efficacy of stent implantation in the treatment of intracranial artery stenosis. Treatment with edaravone alleviated depression in patients with symptomatic intracranial artery stenosis and reduced the incidence of PSD.^[14]^

## Acknowledgments

We would like to thank Qun Wang (Beijing Tiantan Hospital, Capital Medical University, Beijing, China) for editing the manuscript for English grammar.

## Author contributions

**Conceptualization:** Zhaohui Zhang, gaohua wang.

**Data curation:** zhaohong kong, jian jiang.

**Formal analysis:** zhaohong kong, jian jiang.

**Funding acquisition:** Zhaohui Zhang.

**Methodology:** zhaohong kong, jian jiang, ming Deng.

**Project administration:** zhaohong kong.

**Resources:** zhaohong kong, jian jiang.

**Software:** zhaohong kong.

**Supervision:** zhaohong kong, Zhaohui Zhang.

**Writing – original draft:** zhaohong kong.

**Writing – review & editing:** zhaohong kong, Zhaohui Zhang, gaohua wang.

## References

[1] Sacco RL Kargman DE Gu Q Race-ethnicity and determinants of intracranial atherosclerotic cerebral infarction. The Northern Manhattan Stroke Study. Stroke 1995;26:14–20. 7839388 10.1161/01.str.26.1.14

[2] Liu CY Chen CQ Intra- and extracranial atherosclerotic stenosis in China: epidemiology, diagnosis, treatment and risk factors. Eur Rev Med Pharmacol Sci 2014;18:3368–79. 25491610

[3] Huang YN Gao S Li SW Vascular lesions in Chinese patients with transient ischemic attacks. Neurology 1997;48:524–5. 9040750 10.1212/wnl.48.2.524

[4] Kasner SE Chimowitz MI Lynn MJ Predictors of ischemic stroke in the territory of a symptomatic intracranial arterial stenosis. Circulation 2006;113:555–63. 16432056 10.1161/CIRCULATIONAHA.105.578229

[5] Arenillas JF Molina CA Montaner J Progression and clinical recurrence of symptomatic middle cerebral artery stenosis: a long-term follow-up transcranial Doppler ultrasound study. Stroke 2001;32:2898–904. 11739993 10.1161/hs1201.099652

[6] Kern R Steinke W Daffertshofer M Stroke recurrences in patients with symptomatic vs asymptomatic middle cerebral artery disease. Neurology 2005;65:859–64. 16186524 10.1212/01.wnl.0000175983.76110.59

[7] Kasner SE Lynn MJ Chimowitz MI Warfarin vs aspirin for symptomatic intracranial stenosis: subgroup analyses from WASID. Neurology 2006;67:1275–8. 17030766 10.1212/01.wnl.0000238506.76873.2f

[8] Osama O. Zaidat Brian-Fred Fitzsimmons Britton Keith Woodward For the VISSIT trial investigators effect of a balloon-expandable intracranial stent vs medical therapy on risk of stroke in patients with symptomatic. Intracranial stenosis the VISSIT randomized clinical trial. JAMA 2015;313:1240–8. 25803346 10.1001/jama.2015.1693

[9] Bruce CV Campbell MD Peter J Endovascular therapy for ischemic stroke with perfusion-imaging selection. N Engl J Med 2015;372:1009–18. 25671797 10.1056/NEJMoa1414792

[10] Kimura M Robinson RG Kosier JT Treatment of cognitive impairment after poststroke depression: a double-blind treatment trials. Stroke 2000;31:1482–6. 10884441 10.1161/01.str.31.7.1482

[11] Watanabe Tashara M Todo S The novel antioxidant edaravone: from bench to bedside. Cardiovasc Ther 2008;26:101–14. 18485133 10.1111/j.1527-3466.2008.00041.x

[12] Ojagbemi A Owolabi M Atalabi M Stroke lesions and post stroke depression among survivors in Ibadan, Nigeria. Afr J Med Sci 2013;42:245–51. 24579386

[13] Park SI Kim JH Kwak JK Intracranial stenting for severe symptomatic stenosis: self-expandable versus balloon-expandable stents. Interv Neuroradiol 2013;19:276–82. 24070075 10.1177/159101991301900303 PMC3806001

[14] Gao P Zhao Z Wang D China Angioplasty and Stenting for Symptomatic Intracranial Severe Stenosis (CASSISS): a new, prospective, multicenter, randomized controlled trial in China. Interv Neuroradiol 2015;21:196–204. 25934656 10.1177/1591019915581778 PMC4757239

[15] Golimbet VE Volel BA Kopylov FY Anxiety and polymorphism Val66Met of BDNF gene—predictors of depression severity in ischemic heart disease. Kardiologiia 2015;55:9–13. 28294821

[16] Min SI Yoon KC Min SK Current strategy for the treatment of symptomatic spontaneous isolated dissection of superior mesenteric artery. J Vasc Surg 2011;54:461–6. 21571493 10.1016/j.jvs.2011.03.001

[17] Lim S Moon MK Shin H Effect of S-adenosylmethionine on neointimal formation after balloon injury in obese diabetic rats. Cardiovasc Res 2011;90:383–93. 21245056 10.1093/cvr/cvr009

[18] Sierra C Cerebral small vessel disease, cognitive impairment and vascular dementia. Panminerva Med 2012;54:179–88. 22801435

[19] Matsumoto S Murozono M Kanazawa M Edaravone and cyclosporine A as neuroprotective agents for acute ischemic stroke. Acute Med Surg 2018;5:213–21. 29988669 10.1002/ams2.343 PMC6028804

[20] Edaravone Acute Infarction Study Group. Effect of a novel free radical scavenger, edaravone (MCI-186), oil acute brain infarction. Randomized, placebo controlled, double-blind study at Multi-centers. Cerebrovasc Dis 2003;15:222–9. 12715790 10.1159/000069318

[21] Mazure CM Weinberger AH Pittman B Gender and stress in predicting depressive symptoms following stroke. Cerebrovasc Dis 2014;38:240–6. 25401293 10.1159/000365838 PMC4283501

[22] McCarthy MJ Sucharew HJ Alwell K Age, subjective stress, and depression after ischemic stroke. J Behav Med 2016;39:55–64. 26245159 10.1007/s10865-015-9663-0 PMC4724284

[23] Wang P Cao J Liu N Protective effects of edaravone in adult rats with surgery and lipopolysaccharide administration-induced cognitive function impairment. PLoS One 2016;11:e0153708. 27116382 10.1371/journal.pone.0153708 PMC4846078

[24] Jangra A Sriram CS Dwivedi S Sodium phenylbutyrate and edaravone abrogate chronic restraint stress-induced behavioral deficits: implication of oxido-nitrosative, endoplasmic reticulum stress cascade, and neuroinflammation [J]. Cell Mol Neurobiol 2017;37:65–81. 26886752 10.1007/s10571-016-0344-5 PMC11482225

[25] Leonard BE Inflammation and depression: a causal or coincidental link to the pathophysiology? Acta Neuropsychiatr 2018;30:1–6. 10.1017/neu.2016.69 28112061

[26] Chen CY Chen CL Yang YH Poststroke depressive symptoms are associated with increased oxidative deoxyribonucleic acid damage. J Neuropsychiatry Clin Neurosci 2018;30:139–44. 29366374 10.1176/appi.neuropsych.17050108

[27] Rohr UD The impact of testosterone imbalance on depression and women's health. Maturitas 2002;41:25–46. 10.1016/s0378-5122(02)00013-0 11955793

[28] Carod-Artal FJ Trizotto DS Coral LF Determinants of quality of life in Brazilian stroke survivors. J Neurol Sci 2009;284:63–8. 19411080 10.1016/j.jns.2009.04.008

[29] 1993;Pepper GM Koenigsberg R Zito JL Alteration of serum pituitary hormone levels in postmenopausal women with stroke. stroke. 24:805–8. 10.1161/01.str.24.6.805 8099454

[30] Mcintyre RS Mancini D Eisfeld BS Circulated bioavailable testosterone levels and depression in middle-aged men. Psychoneuroendocrinology 2006;31:1029–35. 16908107 10.1016/j.psyneuen.2006.06.005

[31] Muck-Seler D Pivace N Mustapic M Platelet serotonin and plasma prolactin and cortisol in healthy, depressed and schizophrenic women. Psychiatry Res 2004;127:217–26. 15296821 10.1016/j.psychres.2004.04.001

[32] Schmidt PJ Nieman L Danaceau MA Estrogen replacement in perimenopause-related depression: a preliminary report. Am J Obstet Gynecol 2000;183:414–20. 10942479 10.1067/mob.2000.106004

[33] Carrasco GA Barker SA Zhang Y Estrogen treatment increases the levels of regulator of G protein signaling-Z1 in the hypothalamic paraventricular nucleus: possible role in desensitization of 5-hydroxy tryptamine A receptors. Neuroscience 2004;127:261–7. 15262317 10.1016/j.neuroscience.2004.05.031

[34] Doncarlos L Muma NA Battaglia G Estrogen desensitizes 5-HT (l A) receptors and reduces levels of G (z), G(il) and G (i3) proteins in the hypothalamus. Neuropharmacology 2000;39:1823–32. 10884563 10.1016/s0028-3908(99)00264-6

[35] Shores MM Moceri VM Sloan KL Low testosterone levels predict incident depressive illness in older men: effects of age and medical morbidity. J Clin Psychiatry 2005;66:7–13. 10.4088/jcp.v66n0102 15669883

